# Possibilities and pitfalls in quantifying the extent of cysteine sulfenic acid modification of specific proteins within complex biofluids

**DOI:** 10.1186/1471-2091-11-25

**Published:** 2010-07-01

**Authors:** Douglas S Rehder, Chad R Borges

**Affiliations:** 1 Molecular Biomarkers, The Biodesign Institute at Arizona State University, Tempe, AZ 85287, USA

## Abstract

**Background:**

Cysteine sulfenic acid (Cys-SOH) plays important roles in the redox regulation of numerous proteins. As a relatively unstable posttranslational protein modification it is difficult to quantify the degree to which any particular protein is modified by Cys-SOH within a complex biological environment. The goal of these studies was to move a step beyond detection and into the relative quantification of Cys-SOH within specific proteins found in a complex biological setting--namely, human plasma.

**Results:**

This report describes the possibilities and limitations of performing such analyses based on the use of thionitrobenzoic acid and dimedone-based probes which are commonly employed to trap Cys-SOH. Results obtained by electrospray ionization-based mass spectrometric immunoassay reveal the optimal type of probe for such analyses as well as the reproducible relative quantification of Cys-SOH within albumin and transthyretin extracted from human plasma--the latter as a protein previously unknown to be modified by Cys-SOH.

**Conclusions:**

The relative quantification of Cys-SOH within specific proteins in a complex biological setting can be accomplished, but several analytical precautions related to trapping, detecting, and quantifying Cys-SOH must be taken into account prior to pursuing its study in such matrices.

## Background

A rapidly expanding body of evidence demonstrates that cysteine sulfenic acid (Cys-SOH) formation within protein molecules can serve as a means of regulating protein activity. Under variant biochemical circumstances it may serve to mediate redox signaling [1-5], fundamentally alter protein activity [1,2,4-13], or absorb and deflect oxidative insults [1,10,11,14-20]. Much of this evidence has only recently come to light because the unstable, transient nature of Cys-SOH has prevented its thorough study in decades past: As a protein modification it is generally unstable unless enveloped within a stabilizing protein microenvironment.

The ability to quantify the extent to which individual proteins are modified by Cys-SOH in complex biological matrices is an important step in understanding the full range of biological processes in which the posttranslational modification plays a role. Naturally, complex biological samples represent the ultimate context for such relative-percent-abundance (RPA) determinations of protein Cys-SOH. But a combined lack of spectral absorption properties and chemical instability have historically left few options available with regard to analyzing protein Cys-SOH in even the simplest matrix. Covalent trapping with a sulfenic acid-specific probe that changes an intrinsic property (e.g., mass or optical absorbance characteristics) of labeled protein molecules is, currently, the only viable path of routine detection. To date, the most commonly used chemical traps of Cys-SOH are molecules with dimedone or sulfhydryl functional groups [3,6,21-24]. These have been employed successfully and are quite useful in the analysis of isolated proteins; the former have even been applied to detecting the presence of protein Cys-SOH in biological samples [5,25-28]. But the molecular complexity of biological samples imparts a layer of analytical difficulty that has yet to be overcome with regard to determining the precise degree to which specifically targeted proteins are modified by Cys-SOH in their native environments. Thus, beyond simple (positive or negative) detection of Cys-SOH within specific proteins or the relative quantification of Cys-SOH in many proteins together in bulk, little has been developed in the way of technologies to quantify the precise degree to which particular proteins within complex biological samples are modified by Cys-SOH.

Herein we report on the application of mass spectrometric immunoassay (MSIA) [29-34] to the detection and relative quantification of Cys-SOH as it modifies specifically targeted proteins in human plasma. MSIA is high throughput affinity chromatography, followed by the analysis of intact proteins by mass spectrometry, using relative mass spectral peak intensities corresponding to modified and unmodified protein forms as representative of their relative abundances [35]. This is a well established analytical procedure [29,32,33,36-42] and is analogous to an ultra-high resolution, semiquantitative western blot. In addition to the now well known example of albumin [14,15,24,43,44] we report on the RPA of previously undocumented Cys-SOH formation in transthyretin (TTR) as it occurs within the biological matrix of human plasma. Taken altogether, however, our results also compel the description of several analytical precautions related to trapping, detecting, and quantifying Cys-SOH in complex biological samples.

## Results and Discussion

*Detection and Relative Quantification of Cys-SOH Formation within Specific Plasma Proteins*: The strategy taken was to trap Cys-SOH, then extract and analyze all molecular forms of targeted proteins using MSIA. Albumin and TTR serve as ideal model proteins by which to assess this strategy because each possesses a single free cysteine residue--meaning that only one mole of Cys-SOH can form per mole of protein.

**Figure 1:**
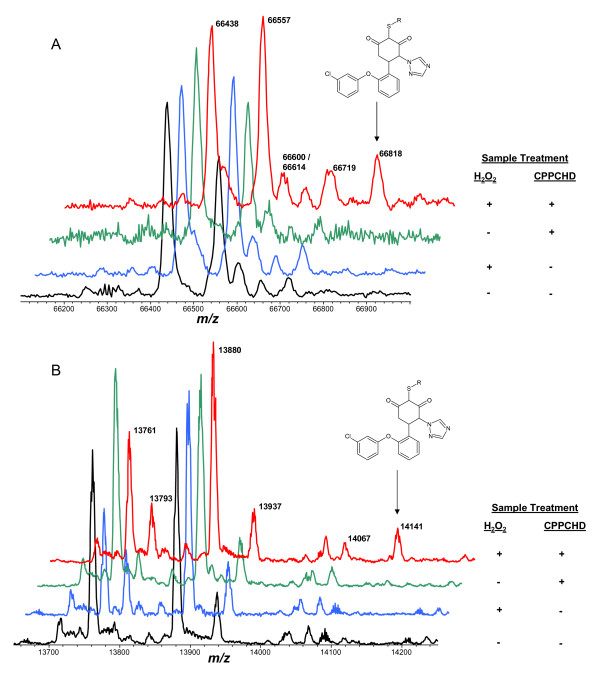
**Mass spectra of A) albumin and B) TTR extracted from human plasma that was treated with/without hydrogen peroxide and with/without the dimedone-based Cys-SOH trapping reagent CPPCHD**. For albumin (A), deconvoluted "MH^+^" peaks are assigned as follows: 66438 = Native/unmodified (Calc. 66439), 66557 = S-Cysteinylated (Calc. 66558), 66600 = Glycated native form (Calc. 66601), 66614 = S-CysGly (Calc. 66615), 66719 = Glycated S-Cysteinylated form (Calc. 66720), 66818 = CPPCHD-modified native form (Calc. 66819). For TTR (B), 13761 = Native/unmodified (Calc. 13762), 13793 = Cys-sulfinic acid (SO_2_H) due to over oxidation (Calc. 13794), 13880 = S-Cysteinylated (Calc. 13881), 13937 = S-CysGly (Calc. 13938), 14067 = S-Glutathionylated (Calc. 14069), 14141 = CPPCHD-modified native form (Calc. 14142). For TTR, partial peak splitting is due to incomplete monoisotopic resolution. Assigned "MH^+^" values in deconvoluted spectra are centroided average masses.

Albumin and TTR were immunoaffinity-extracted from human plasma that was treated with hydrogen peroxide and the dimedone-based Cys-SOH trapping reagent 5-[2-(3-chlorophenoxy)phenyl]-4-(1H-1,2,4-triazol-1-yl)-1,3-cyclohexanedione (CPPCHD; Figure 1). Dimedone itself and other dimedone-based probes such as DCP-Bio1 [23] produce qualitatively analogous results. The high mass of dimedone-based probes such as CPPCHD and DCP-Bio1 provides a significant mass shift to the protein such that tagged protein molecules lie out of the mass range of commonly observed posttranslationally modified protein variants. For example, S-cysteinylation (Δm +119 Da) and S-Cysteinylglycinylation (Δm +176 Da) are frequently observed as posttranslationally modified forms of free cysteine-containing proteins (Figure 1, [32,37]). The RPA of each posttranslationally modified form of the proteins analyzed in Figure 1 are listed in Table 1.

**Table 1:** Relative percent abundance (RPA) data for the mass spectra in Figures 1 and 4

**Figure 1a -- Albumin**								
m/z 66438	m/z 66470	m/z 66557	m/z 66600 & 66614	m/z 66655	m/z 66719	m/z 66818	Sample Treatment	

RPA Native	RPA SO_2_H	RPA S-Cys	RPA Native Glycated & S-CysGly	RPA NonCov. Phosphate Adduct	RPA S-Cys + Glycation	RPA CPPCHD Adduct	H_2_O_2_	CPPCHD

32%	10%	38%	6%	3%	5%	6%	+	+

51%	0%	34%	9%	1%	5%	0%	-	+

32%	12%	43%	6%	3%	4%	0%	+	-

49%	0%	36%	6%	3%	5%	0%	-	-

								

**Figure 1b -- TTR**								

m/z 13761	m/z 13793	m/z 13880	m/z 13937	m/z 14067	m/z 14141		Sample Treatment	

RPA Native	RPA SO_2_H	RPA S-Cys	RPA S-CysGly	RPA S-Glutathione	RPA CPPCHD Adduct		H_2_O_2_	CPPCHD

23%	9%	48%	13%	3%	3%		+	+

39%	3%	46%	10%	3%	0%		-	+

21%	12%	52%	12%	3%	0%		+	-

39%	2%	48%	10%	1%	0%		-	-

								

**Figure 4a -- Albumin**								

m/z 66438	m/z 66470	m/z 66557	m/z 66600 & 66614	m/z 66636	m/z 66655	m/z 66719	Sample Treatment	

RPA Native	RPA SO_2_H	RPA S-Cys	RPA Native Glycated & S-CysGly	RPA TNB Adduct	RPA NonCov. Phosphate Adduct	RPA S-Cys + Glycation	H_2_O_2_	TNB

32%	7%	37%	3%	15%	3%	4%	+	+

49%	0%	37%	5%	0%	4%	5%	-	+

32%	13%	42%	5%	0%	3%	4%	+	-

48%	0%	38%	7%	0%	3%	4%	-	-

								

**Figure 4b -- TTR**								

m/z 13761	m/z 13793	m/z 13880	m/z 13937	m/z 13959	m/z 14067		Sample Treatment	

RPA Native	RPA SO_2_H	RPA S-Cys	RPA S-CysGly	RPA TNB Adduct	RPA S-Glutathione		H_2_O_2_	TNB

19%	11%	48%	11%	7%	4%		+	+

29%	IP	50%	9%	8%	4%		-	+

21%	12%	52%	12%	0%	3%		+	-

38%	2%	46%	12%	0%	2%		-	-

IP = "Interfering Peak", cannot properly integrate

Because it can readily be analyzed intact by ESI-MS after a simple ~1000-fold dilution of plasma, albumin serves as a convenient model protein by which to assess the accuracy and precision of these techniques for relative quantification of Cys-SOH-modified protein. By comparing the RPA of Cys-SOH modified albumin detected following plasma preparation by dilution vs. immunoaffinity extraction, it can be determined whether or not antibodies bind dimedone-tagged forms of the proteins with different affinity than that with which they bind naturally occurring form(s). (By the term "dimedone-tagged" we refer to molecules that bear a 1,3-cyclohexanedione (dimedone-like) functional group.)

**Figure 2:**
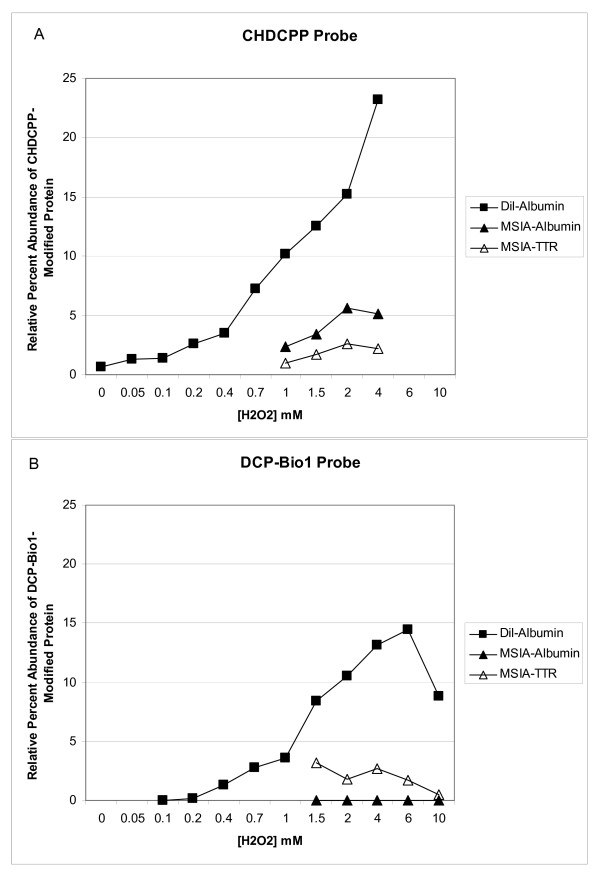
**Relative percent abundance (RPA) of cysteine sulfenic acid on albumin (solid markers) as reported by the A) CPPCHD and B) DCP-Bio1 Cys-SOH probes over a range of plasma concentrations of hydrogen peroxide**. As shown for albumin, there is a significant difference in the percent of modified protein detected when samples are prepared by dilution vs. MSIA. This demonstrates relative antibody aversion for CPPCHD and DCP-Bio1-tagged forms of albumin. Additionally, the RPA values for cysteine sulfenic acid-modified albumin appear significantly lower when trapped by (higher concentrations of) DCP-Bio1--demonstrating an empirical discrepancy in Cys-SOH trapping efficiency between the two dimedone-based probes. Plasma from the same individual was used for collection of these datasets. Data on the RPA of dimedone-tagged TTR (hollow markers) are shown for reference. RPA values decrease at the highest concentrations of hydrogen peroxide due to overoxidation of Cys-SH to cysteine sulfinic acid (SO_2_H). Evidence to support this assertion can be seen as a +32 Da mass shift from the Cys-SH form (and only the Cys-SH form) of the hydrogen peroxide treated samples in **Figure 1, panel B**.

To begin to assess the accuracy and precision of the technique for determining the RPA of Cys-SOH modified proteins, a series of experiments were run in which human plasma was exposed to increasing concentrations of hydrogen peroxide to generate abundant quantities of Cys-SOH. Plots of the RPA of dimedone-tagged target protein vs. hydrogen peroxide concentration (Figure 2) reveal two potential problems. First, by comparing the RPA (y-axis) of tagged albumin when analyzed by dilution vs. MSIA (for both dimedone-based probes; Figure 2, Panels A and B), a quantitative discrepancy is readily apparent in which the MSIA-based technique reports a lower RPA of covalently tagged albumin than the dilution technique. The most likely explanation for this phenomenon is that there is a diminished binding affinity of the anti-albumin antibodies for tagged forms of the protein. Second, by comparing the RPA of Cys-SOH modified albumin reported by the two different dimedone-based probes across a series of hydrogen peroxide concentrations (Figure 2, Panel A vs. B, solid squares), a substantial quantitative discrepancy is readily apparent: DCP-Bio1 was present in solution at a higher concentration than CPPCHD but results in lower apparent values for RPA of Cys-SOH. This is most likely due to differences in reaction rates between the two probes for the Cys-SOH site. This brings up the question of which, if either probe reports a truly accurate (surrogate) quantitative value for the RPA of Cys-SOH originally present in the plasma.

The final calculated concentrations of CPPCHD and DCP-Bio1 in solution were 9 mM and 17 mM, respectively. However it is possible that these final concentrations may not have actually been reached due to solubility issues: CPPCHD has a calculated (ALOGpS [45]) aqueous solubility of about 25 μM and DCP-Bio1 of about 120 μM. However, the existence of 5-10% DMSO in solution coupled with evidence from others that DCP-Bio1 shows increased rates of alkylation of pure protein with increasing concentrations of DCP-Bio1 up to about 1 mM [23,46], leads to the final conclusion that both probes were saturated in solution and that the concentration of DCP-Bio1 was higher than that of CPPCHD.

But even at saturating concentrations, the rates of reaction of dimedone-based probes with Cys-SOH are slow enough that, in a complex biological fluid like human plasma, resulting reports of Cys-SOH RPA stand to be in error because of the presence of endogenous free small molecule thiols such as cysteine, homocysteine, and glutathione which react much faster with protein Cys-SOH than dimedone [24]. The concentration of *free *cysteine in human plasma is typically in the 1-10 μM range [47], if not more [47-49]. Experimentally, evidence for the competitive reaction of endogenous free cysteine with protein Cys-SOH can readily be seen vis-à-vis an increase in the intensity of the peaks representing S-cysteinylated albumin and TTR in samples to which hydrogen peroxide was added to generate Cys-SOH (Figure 1 and Table 1). This is a fundamental limitation of dimedone-based probes of protein Cys-SOH.

By nature no surrogate trapping technique can guarantee a quantitatively perfect representation of a trapped molecular species--particularly when the species to be trapped are molecularly unstable and there is endogenous competition for reaction with them. This suggests that, other factors held equal, the trapping reagent with the fastest reaction rate toward the unstable target molecule will provide the most quantitatively accurate estimate of the unstable species.

### Assessment of 2-nitro-5-thiobenzoic acid as a Quantitative Probe

2-nitro-5-thiobenzoic acid (a.k.a., thionitrobenzoic acid or TNB) is the disulfide-reduced form of Ellman's reagent which is classically used for the determination of free thiol groups in proteins. Recently, Turell et al determined that the rate constant for TNB reaction with albumin Cys-SOH is 105 M^-1 ^s^-1^--over 3000 times greater than that for unsubstituted dimedone and 5 times greater than that for free cysteine [24]. Thiols do not spontaneously react with other thiols in the absence of an oxidizing agent and, under the conditions employed, TNB does not readily participate in intramolecular disulfide exchange by reducing/inserting itself into the existing disulfide bonds of albumin. When this is the case, neither reduced thiols nor disulfides interfere with TNB as a probe of Cys-SOH [24]. Under the conditions used for the experiments described below, no albumin modified by two or more molecules of TNB was detected. This serves as experimental verification that TNB was not reacting with protein disulfide bonds. (Though not applicable to these studies, however, it is possible for protein-TNB mixed disulfides to be removed by intramolecular disulfide exchange wherein a molecule of TNB is released and a disulfide bond is formed between two cysteine residues that normally interact with one another as part of normal protein operation [50].)

**Figure 3:**
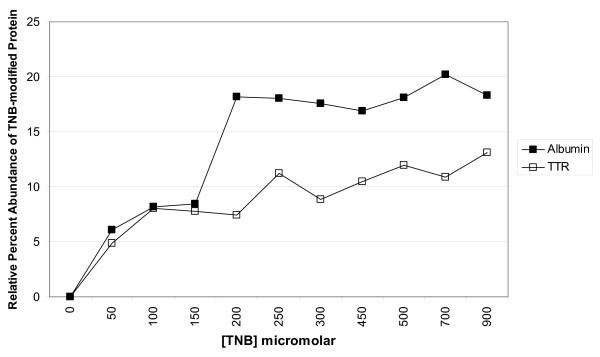
**Relative percent abundance (RPA) of cysteine sulfenic acid on albumin (solid markers) and TTR (hollow markers) as reported by increasing concentrations of TNB**. Plasma samples were prepared by oxidation with 4 mM hydrogen peroxide for 2 minutes at 37°C, followed by quenching with catalase, addition of TNB, immediate removal of TNB via gel filtration spin columns, 1000-fold dilution of plasma, and analysis by ESI-MS.

To assess the utility of TNB as a quantitative probe for the RPA of Cys-SOH-modified albumin and transthyretin in human plasma, TNB was added to plasma at increasing concentrations immediately after the plasma was exposed to 4 mM hydrogen peroxide (for 2 minutes) then catalase. As described below, *immediate *protein purification and analysis (i.e., *zero *explicit TNB incubation time) was found to be necessary to minimize artifactual formation of Di-TNB (Ellman's reagent/DTNB) and subsequent labeling of free cysteine residues. As such, it was also important to minimize the concentration of TNB added to plasma samples. At concentrations beyond 250 μM the addition of TNB did not result in additional labeling of protein Cys-SOH (Figure 3); thus this concentration of TNB was employed in subsequent experiments.

**Figure 4:**
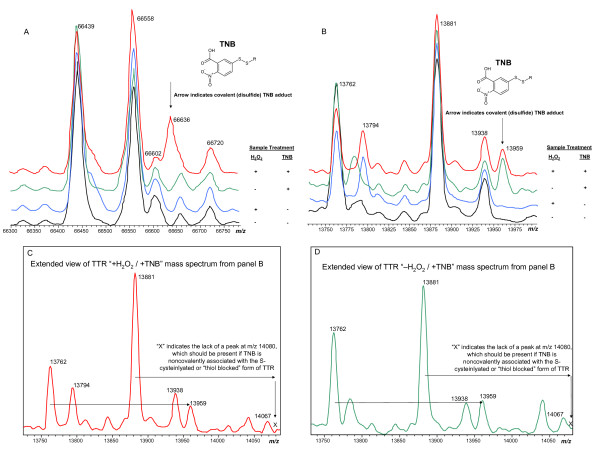
**Mass spectra of A) albumin and B) TTR extracted from human plasma that was treated with/without hydrogen peroxide and with/without the Cys-SOH trapping reagent TNB**. For albumin (A), deconvoluted "MH^+^" peaks are assigned as follows: 66439 = Native/unmodified (Calc. 66439), 66558 = S-Cysteinylated (Calc. 66558), 66602 = Glycated native form (Calc. 66601), 66636 = TNB-adducted native form (Calc. 66636), 66720 = Glycated S-Cysteinylated form (Calc. 66720). For TTR (B), 13762 = Native/unmodified (Calc. 13762), 13794 = Cys-sulfinic acid (SO_2_H) due to over oxidation (Calc. 13794), 13881 = S-Cysteinylated (Calc. 13881), 13938 = S-CysGly (Calc. 13938), 13959 = TNB-adducted native form (Calc. 13959). Assigned "MH^+^" values in deconvoluted spectra are centroided average masses. All samples were immediately purified with a gel filtration spin column upon addition of TNB and further prepared as quickly as possible for introduction into the mass spectrometer (~ 3 minutes for diluted samples and <15 minutes for MSIA samples). Albumin was prepared by dilution and TTR was prepared by MSIA (with the exception of the - H_2_O_2_/^+^ TNB sample for TTR which was prepared by dilution to facilitate the fastest possible introduction into the mass spectrometer). Panels C and D provide extended views of the samples to which TNB was added, demonstrating that TNB reacts only with the form of TTR which originally carried a free thiol. As illustrated, TNB does not bind noncovalently to S-cysteinylated or "thiol blocked" TTR.

Under these conditions TNB, like dimedone-based probes, reacts specifically with Cys-SOH modified protein molecules (Figure 4). (RPA data of the different protein forms can be seen in Table 1.) Interestingly, based on its spontaneous reactivity with TNB in the absence of exogenous hydrogen peroxide (Figure 4 Panel B, - H_2_O_2_/+ TNB trace), a significant proportion of TTR protein molecules appear to possess native Cys-SOH. This finding is not due to artifactual non-covalent association of TNB with TTR. TNB only binds to the native, free cysteine-containing form of TTR and not the thiol-blocked S-cysteinylated form (Figure 4, Panels C and D).

**Figure 5:**
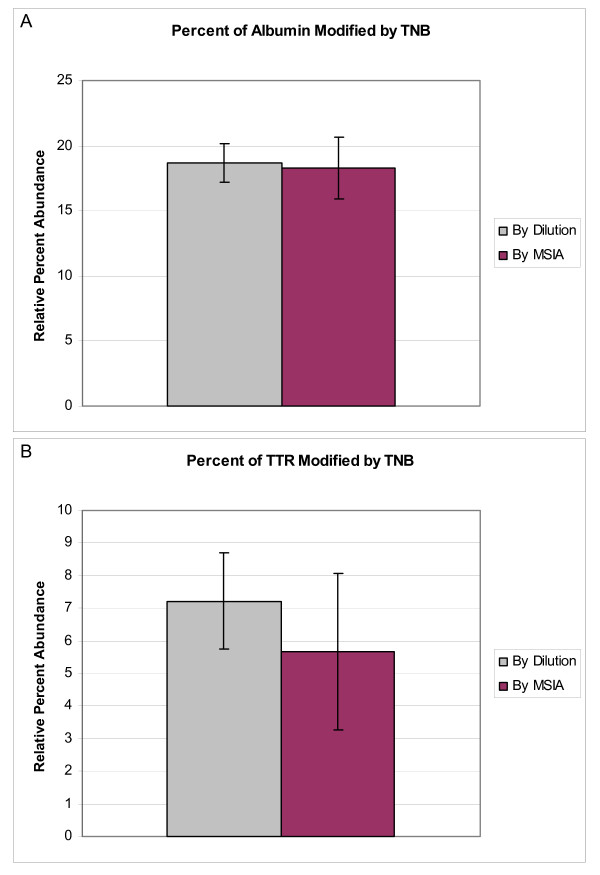
**Precision studies for the use of TNB (250 μM) as a trap of Cys-SOH in A) albumin and B) TTR, prepared from oxidized human plasma by dilution and by MSIA**. Each column represents the average ± standard error of 5 independent samples. No statistically significant differences were noted for dilution vs. MSIA.

**Figure 6:**
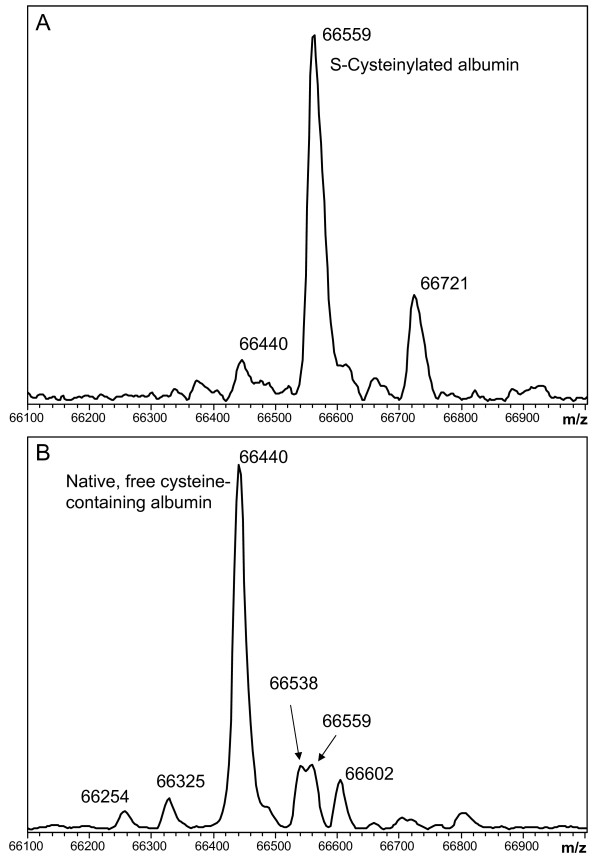
**Illustration of the wide range of relative percent abundances of Native and S-Cysteinylated albumin found in the human population**. Data were acquired from human plasma obtained from A) an individual with congestive heart failure, diabetes, and a history of myocardial infarction (4.5% Native form), and B) a healthy individual (66% Native form). Deconvoluted "MH^+^" peaks are assigned as follows: 66254 = N-terminal truncation (-DA) (Calc. 66253), 66325 = N-terminal truncation (-D) (Calc. 66324), 66440 = Native/unmodified (Calc. 66439), 66538 = Non-covalent phosphate adduct (plasma anticoagulant artifact) (Calc. 66537), 66559 = S-Cysteinylated (Calc. 66558), 66602 = Glycated native form (Calc. 66601), 66721 = Glycated S-Cysteinylated form (Calc. 66720).

To assess analytical precision and the potential for antibody bias against TNB-labeled protein, five replicate plasma aliquots were incubated with hydrogen peroxide/catalase and 250 μM TNB, then analyzed for TNB-modified albumin and TTR by both dilution and (as separate samples) MSIA. For both albumin and TTR there was no statistically significant difference in the RPA of TNB-modified protein when samples were prepared by dilution vs. immunoaffinity extraction (Figure 5). Though, for TTR, a t-test p-value of just over 0.06 suggests that there may indeed be a slight antibody bias against TNB-labeled protein molecules. For plasma prepared by dilution, the mean relative percent TNB-tagged albumin was 19 ± 1.5 SE; by MSIA it was 18 ± 2.4 SE. For TTR, these values were 7.2 ± 0.18 SE and 5.7 ± 1.2 SE, respectively. These values have not previously been reported for TTR because the existence of Cys-SOH modified TTR was unknown. However, for albumin, our results are consistent with a previously reported value (18%) for percent Cys-SOH modification under similar oxidative conditions but in the absence of a complex biological matrix such as plasma [24]. Notably, these values may well vary depending on the percentage of albumin molecules carrying a free cysteine residue. During the course of examining hundreds of individual plasma samples, we have observed the percentage of free cysteine-containing albumin to range from less than 5% to greater than 65% (Figure 6).

### Analytical Precautions for the Use of TNB

When exposed to air for prolonged periods of time, TNB can form disulfide homodimers--resulting in the formation of DTNB which readily reacts with free cysteine residues. As such, when employed as a trap of Cys-SOH without precaution to prevent such homodimer formation, erroneously high values for relative percent Cys-SOH abundance may be obtained.

To quantitatively determine the extent of this effect at 250 μM TNB, a fresh solution of TNB was prepared in a 25 mM bicarbonate buffer containing 0.15 M NaCl (pH 7.4) and monitored for loss of absorbance at 412 nm [24,51] over 15 minutes--a time period longer than that required to immediately pass TNB-exposed plasma through a gel filtration spin column (removing > 95% of the TNB), dilute or immunoaffinity extract, and introduce the resulting sample into a mass spectrometer. (The molar absorptivity at 412 nm for DTNB is 211 M^-1 ^cm^-1 ^and that for TNB is 14,150 M^-1 ^cm^-1 ^[51].) Decay was limited to a loss of 0.5 μM/min, corresponding to a potential *maximum *DTNB formation rate of 0.25 μM/min. Conservative (i.e., overly abundant) incubations of 1-10 μM DTNB in plasma for 25 minutes showed that this rate of formation of DTNB from TNB results in no detectable (i.e., less than ~ 1% relative abundance) formation of TNB-modified albumin and only trace/marginally detectable quantities of TNB-modified TTR. But given that, in our experiments, freshly reduced TNB was employed (see Methods section) and >95% of TNB was filtered out of our samples within seconds via centrifugal gel filtration, it is reasonable to assert that artifactual formation of DTNB and its subsequent contribution to our measured RPA values of TNB-modified protein was negligible.

### Advantages and Disadvantages of Cys-SOH Relative Quantification by Mass Spectrometric Analysis of Intact Proteins

When coupled with a rapid purification technique, mass spectral analysis of intact proteins provides a degree of molecular detail and an ability to analyze proteins in complex biological matrices that is essentially impossible to match with other available techniques such as spectrophotometry or western blotting. For example, the mass spectral data shown in Figures 4c and 4d readily reveal that *only *the protein molecules with an available free cysteine residue are modified by TNB (whether or not H_2_O_2 _was used as an oxidant). Protein molecules in which the single free cysteine residue was already S-cysteinylated remained unmodified.

A potential criticism of the technique is that possible differences in ionization efficiency between differentially modified protein molecules may render relative quantification of the different protein forms inaccurate--i.e., that resulting mass spectral integrals may not represent the *exact *molar percentage of each protein variant detected. However, considering the overall size of proteins and the sheer number of charges on each protein molecule (+8 to +19 for TTR and +30 to +66 for albumin) coupled with the at-most ± 1 charge-unit effect of a single differential substituent on whole protein ionization, the argument for an effect of ionization efficiency on apparent molar abundances becomes negligible.

### Physiological Relevance

With few exceptions, most studies of protein-SOH described in the literature involve induction of protein-SOH using non-physiological concentrations of exogenous oxidants such as H_2_O_2_. Because the addition of exogenous oxidants to create protein-SOH is required for most proteins, the RPAs of protein-SOH observed in this and other studies reported in the literature are certainly higher than might be found physiologically. However, this study, as well as others, clearly shows that mixed protein-glutathione and protein-cysteine disulfides exist naturally in significant quantities. Based on the observations reported in this study, we hypothesize that a major physiological role of protein-SOH (for the vast majority of proteins) is to serve as an oxidative intermediate in the formation of reversible mixed protein disulfides. As such, the *in vivo *RPA of protein-SOH in most proteins at any given instant in time will likely be low.

## Conclusions

Molecular probes which covalently trap Cys-SOH can yield modified proteins that are less well-recognized by antibodies than their unmodified/natural counterparts. Each combination of protein, probe, and antibody stands to differ in this effect and must be investigated empirically. Dimedone-based probes of Cys-SOH are useful for detecting the presence of protein-specific Cys-SOH within complex biological matrices [5,25-28], but with regard to relative quantification, their reaction rates with Cys-SOH may be outpaced by reactions with endogenous thiols. TNB reacts faster with Cys-SOH than endogenous thiols or dimedone [24]; we have shown herein that when proper precautions are taken to ensure its stability, TNB's fast reaction rate, limited antibody bias, and net mass shift make it useful in the relative quantification of Cys-SOH on specific proteins within human plasma when mass spectrometry is employed for detection. Transthyretin in its native environment appears to be modified by Cys-SOH in the absence of exogenous oxidants. Amongst different individuals, free cysteine-containing plasma proteins can vary widely in the fraction of protein molecules carrying free thiol groups. Thus ultimately, to understand and properly interpret the relative quantification of protein modification by Cys-SOH, it is necessary to be aware of this percentage of target protein molecules that contain free thiol groups and thus are available for modification by Cys-SOH.

## Methods

### Materials

Human plasma from a single healthy female donor and a single healthy male donor was purchased from ProMedDx. Plasma samples were collected offsite under IRB approval and, prior to purchase, were publicly available. Plasma samples were received with all personal identifiers removed, allowing the authors to operate under human subjects "Exemption 4" classification pursuant to Federal regulations 45 CFR Part 46.101(b)(4) (Arizona State University IRB Protocol #: 0705001851, approved 18 May, 2007). Plasma was stored at -80°C until analysis. MSIA pipette tips containing frits with a carboxydextran surface were obtained from Intrinsic Bioprobes, Inc. (Tempe, AZ). Affinity purified polyclonal anti-human serum albumin (Cat. No. A0001) and anti-human transthyretin (Cat. No. A0002) were obtained from DAKO (Carpinteria, CA). 5-[2-(3-chlorophenoxy)phenyl]-4-(1H-1,2,4-triazol-1-yl)-1,3-cyclohexanedione (CPPCHD) was purchased from Ryan Scientific. DCP-Bio1 was generously supplied by Dr. Bruce King of Wake Forest University. Premixed 0.6 M sodium citrate/0.1 M sodium carbonate (pH 9) buffer packets (Cat. No. 28388), 0.5 mL Zeba™ desalting gel filtration spin columns (MWCO = 7,000 Da; Cat. No. 89883) and immobilized tris(carboxyethyl) phosphine disulfide reducing gel (Cat. No. 77712) were acquired from Pierce. HEPES buffered saline (10 mM HEPES, 0.15 M NaCl, pH 7.4; a.k.a. HBS-N) was purchased from GE Healthcare Life Sciences. Five-milliliter HiTrap Q^® ^ion exchange cartridges (Cat No. 54816) and all other chemicals and reagents were from Sigma-Aldrich.

### TNB Synthesis, Purification, Storage and Use

Thionitrobenzoate (TNB) was synthesized by reduction of 5,5'-Dithiobis(2-nitrobenzoic acid) (a.k.a. Ellman's reagent/DTNB) and purified as described elsewhere [24]. Briefly, 40 mL of a 5 mM solution of DTNB was made by alkalinizing a DTNB suspension with NaOH until it completely dissolved. The final pH was 8.5 - 9. A 40-fold excess of β-mercaptoethanol (630 μL) was mixed with the DTNB and allowed to react for 45 minutes at room temperature. Resulting TNB was purified over a 5-mL HiTrap Q^® ^ion exchange cartridge as follows: The cartridge was prerinsed with 25 mL water, then 25 mL of 50 mM HCl, another 25 mL water, then 30 mL of 20 mM Tris, pH 7.5. After applying the TNB sample, the cartridge was rinsed with 25 mL of 20 mM Tris, pH 7.5, 30 mL water, then eluted with 50 mM HCl. The most intensely yellow-colored 1.5-mL fractions were pooled together. TNB concentration was measured by diluting an aliquot of product 200-fold in 0.6 M citrate/0.1 M carbonate buffer, pH 9 and measuring optical absorbance at 412 nm (at which ε = 14,150 M^-1 ^cm^-1 ^above pH 7.3 [51]). One-half milliliter aliquots were stored frozen at -80°C until use, at which time they were thawed at room temperature over 50 μL of immobilized TCEP. Immediately after thawing, 60 μL of 0.5 M NaOH containing 5 mM DTPA was added and mixed well. During use, aliquots were kept on ice but vortexed and quickly sedimented by brief centrifugation prior to each withdrawal of an aliquot. This procedure was found to keep the concentration of TNB constant for at least 6 hours.

### Immobilization of Antibodies on MSIA Pipette Tips

To prepare MSIA pipette tips for antibody immobilization, the unmodified carboxydextran surfaces were rinsed thoroughly with 0.2 M HCl then acetone using a Beckman Multimek 96 pipetting robot. Tips were subsequently ejected and dried under vacuum. After drying, the tips were activated by exposure to 150 μL of 1,1'-carbonyldiimidazole (50 g/L) in 1-methyl-2-pyrrolidinone for 30 minutes. This was accomplished by repeatedly pipetting (aspirating and dispensing) 100 μL of solution over the tip frits. After two brief rinses in 1-methyl-2-pyrrolidinone, tips were exposed to a 0.05 g/L solution of anti-human albumin or anti-human TTR in 0.1 M MES buffered saline, pH 4.7 by repetitively flowing (aspirating and dispensing 750 times) 50 μL volumes of antibody solution (150 μL/well) through the pipette tips. Tips were subsequently blocked with 1 M ethanolamine, pH 8.5 (400 μL/well; 50 × 150 μL aspirate and dispense steps) then exposed to 60 mM HCl 2*(400 μL/well; 50 × 150 μL aspirate and dispense steps) and equilibrated in HEPES buffered saline (HBS-N) 2*(400 μL/well; 50 × 150 μL aspirate and dispense steps). Anti-human serum albumin and anti-human TTR-linked pipette tips were stored in HBS-N at 4°C until the day of use.

### Trapping Cys-SOH in Plasma Proteins

In most but not all cases, hydrogen peroxide was added to plasma samples to induce formation of protein Cys-SOH. Thus, to 50 μL of plasma was added 1 μL of an appropriate stock concentration of hydrogen peroxide to produce the desired final concentration of H_2_O_2 _(usually 4 mM). In some experiments, reaction volumes were doubled (but concentrations held constant). Stock concentrations of hydrogen peroxide were checked for stability by measuring optical absorbance at 240 nm (at which ε = 43.6 M^-1 ^cm^-1 ^[24]). Unless otherwise indicated, plasma samples were incubated at 37°C for 2 minutes, at which time enough volume of a 2 g/L solution of catalase was added to consume 90% of the hydrogen peroxide in 1 second [24]. Cys-SOH probes were added immediately thereafter. For CPPCHD, 3.8 μL of a 130 mM solution in DMSO was added and incubated at 37°C for 30 minutes. For DCP-Bio1, 3.8 μL of a 250 mM solution in DMSO was added and incubated at 37°C for 30 minutes. Samples were then either diluted 2-fold with HBS-N in preparation for extraction by MSIA or diluted 1000-fold in starting LC/MS mobile phase for direct analysis.

When Cys-SOH was trapped with TNB ~ 1 μL of a 12 mM stock solution of TNB was added to produce the final desired concentration of TNB (usually 250 μM). Because of problems with instability (see Results and Discussion), TNB was allowed no explicit reaction time with plasma proteins, but samples were instead immediately applied to a Zeba™ desalting gel filtration spin column (MWCO = 7,000 Da) following the manufacturer recommendations for removal of small molecules. The high rate constant for reaction of TNB with accessible protein Cys-SOH [24] ensured that TNB was given adequate time to react with protein Cys-SOH, even during this short duration of time. After elimination of small molecules from these plasma samples, they were immediately extracted by MSIA or diluted 1000-fold (in 90/10 water/acetonitrile containing 0.1% formic acid) in preparation for LC-ESI-MS. In the latter case, only ~ 3 minutes were allowed to elapse after introduction of TNB into the sample prior to introduction of the sample into the LC-MS; in the former case of MSIA, <15 minutes was allowed to elapse.

### Plasma Protein Analysis by MSIA

Following a preliminary rinse with fresh HBS-N, anti-human albumin or anti-human TTR-derivatized MSIA pipette tips were used to extract albumin and TTR from plasma samples. This was done by aspirating and dispensing the plasma over the MSIA frit with the aid of an electronic pipettor for 10 minutes. MSIA tips were then washed (by drawing from a fresh reservoir of liquid and dispensing to waste) and eluted as follows: Five cycles of 200 μL of HBS-N, five cycles of 200 μL distilled water, five cycles of 200 uL of 2 M ammonium acetate/acetonitrile (3:1 v/v), ten cycles of 200 uL of distilled water. Elution was accomplished by briefly air-drying the pipette frits then drawing 5 μL of a mixture of 100% formic acid/acetonitrile/distilled water (9/5/1 v/v/v), mixing over the pipette affinity capture frit for 30 seconds, and dispensing into a 96-conical well polypropylene autosampler tray. Frits were then washed with an additional 5.5 μL distilled water which was used to dilute the eluted sample. Five microliters of eluent was then immediately injected onto the LC-ESI-MS.

Unlike absolute quantitative analysis in which percent recovery can be an important analytical parameter, analysis of RPA as carried out here depends primarily on achieving good ion counting statistics in the mass spectrometer. The picomole quantities of protein captured by the MSIA tips are more than sufficient to provide excellent ion counting statistics and signal to noise ratios in the mass spectrometer.

Prior to collecting the data presented in this report, reproducibility of the albumin assay was verified by analyzing albumin in 20 different samples from individual donors run in quadruplicate each. Average CV for the RPA of the native form of albumin was 3.8% and that for glycated albumin was 6.5%. Reproducibility data for the analysis of TTR by MSIA have previously been described [37].

### LC-ESI-TOF Mass Spectrometric Analysis of Albumin and Transthyretin

The analysis of human serum albumin and TTR by reversed phase liquid chromatography electrospray ionization mass spectrometry (LC-ESI-MS) was performed by a trap-and-elute form of LC (rather than traditional LC) on an Eksigent nanoLC*1D LC system. Five-microliter samples were injected by a Spark Holland Endurance autosampler in microliter pick-up mode and loaded onto a protein captrap (polymeric/reversed phase sorbent, Michrom Bioresources, Auburn, CA) configured for unidirectional flow on a 6-port divert valve. Mobile phase A consisted of 90% H_2_O/10% acetonitrile, 0.1% formic acid and was used as a high-flow loading solvent at 10 μL per minute. After 2 minutes, the divert valve position was automatically toggled and flow rate over the protein captrap cartridge changed to 1 μL/min. Flow was then ramped over 8 minutes using a linear gradient from 10% to 90% mobile phase B (100% acetonitrile). The captrap eluate was directed to a Bruker MicrOTOF-Q (Q-TOF) mass spectrometer operating in positive ion, TOF-only mode, acquiring spectra in the *m/z *range of 300 to 3000. ESI settings for the Agilent G1385A capillary microflow nebulizer ion source were as follows: End Plate Offset -500 V, Capillary -4500 V, Nebulizer nitrogen 2 Bar, Dry Gas nitrogen 3.0 L/min at 225°C. Data were acquired in profile mode at a digitizer sampling rate of 2 GHz. Spectra rate control was by summation at 1 Hz.

### Data Analysis for Intact Human Serum Albumin and Transthyretin

Approximately 1-1.5 minutes of recorded spectra were averaged across the chromatographic peak apex of human serum albumin and transthyretin elution. The ESI charge-state envelope was deconvoluted with Bruker DataAnalysis v3.4 software to a mass range of 1000 Da on either side of any identified peak. Deconvoluted spectra were baseline subtracted and all peaks were integrated. Tabulated mass spectral peak areas were exported to a spreadsheet for further calculation and determination of the peak areas of interest relative to all other variant forms of albumin or TTR present in the mass spectrum.

## List of Abbreviations

Cys-SOH: Cysteine sulfenic acid; RPA: relative percent abundance; MSIA: mass spectrometric immunoassay; TTR: transthyretin; CPPCHD: 5-[2-(3-chlorophenoxy)phenyl]-4-(1H-1,2,4-triazol-1-yl)-1,3-cyclohexanedione; TNB: thionitrobenzoic acid; DTNB: 5,5'-Dithiobis(2-nitrobenzoic acid); HBS-N: HEPES buffered saline

## Authors' contributions

DSR participated in experimental design, acquired most of the data, and helped draft the manuscript. CRB participated in experimental design, acquired some of the data and wrote most of the manuscript. All authors read and approved the final manuscript.
